# Performance and Stability of Pearl Millet Varieties for Grain Yield and Micronutrients in Arid and Semi-Arid Regions of India

**DOI:** 10.3389/fpls.2021.670201

**Published:** 2021-05-31

**Authors:** P. Sanjana Reddy, C. Tara Satyavathi, Vikas Khandelwal, H. T. Patil, P. C. Gupta, L. D. Sharma, K. D. Mungra, Sumer P. Singh, R. Narasimhulu, H. H. Bhadarge, K. Iyanar, M. K. Tripathi, Devvart Yadav, Ruchika Bhardwaj, A. M. Talwar, V. K. Tiwari, U. G. Kachole, K. Sravanti, M. Shanthi Priya, B. K. Athoni, N. Anuradha, Mahalingam Govindaraj, T. Nepolean, Vilas A. Tonapi

**Affiliations:** ^1^Indian Institute of Millets Research, Hyderabad, India; ^2^All India Coordinated Research Project on Pearl Millet, Jodhpur, India; ^3^Bajra Research Scheme, College of Agriculture, Mahatma Phule Krishi Vidyapeeth (MPKV), Dhule, India; ^4^Agricultural Research Station, Swami Keshavanand Rajasthan Agriculture University (SKRAU), Bikaner, India; ^5^Rajasthan Agricultural Research Institute, Sri Karan Narendra Agriculture University (SKNAU), Jaipur, India; ^6^Pearl Millet Research Station, Junagadh Agricultural University (JAU), Jamnagar, India; ^7^ICAR-Indian Agricultural Research Institute (IARI), New Delhi, India; ^8^Agricultural Research Station, Acharya NG Ranga Agricultural University (ANGRAU), Ananthapuramu, India; ^9^National Agricultural Research Project, Vasantrao Naik Marathwada Krishi Vidyapeeth (VNMKV), Aurangabad, India; ^10^School of Genetics, Tamil Nadu Agricultural University (TNAU), Coimbatore, India; ^11^College of Agriculture, Rajmata Vijayaraje Scindia Krishi Vishwa Vidyalaya (RVSKVV), Gwalior, India; ^12^Chaudhary Charan Singh Haryana Agricultural University (CCSHAU), Hisar, India; ^13^College of Agriculture, Punjab Agricultural University (PAU), Ludhiana, India; ^14^Agricultural Research Station, University of Agricultural Sciences (UAS), Raichur, India; ^15^Zonal Agricultural Research Station, Rajmata Vijayaraje Scindia Krishi Vishwa Vidyalaya (RVSKVV), Morena, India; ^16^Agricultural Research Station, Mahatma Phule Krishi Vidyapeeth (MPKV), Niphad, India; ^17^Regional Agricultural Research Station, Professor Jayashankar Telangana State Agricultural University (PJTSAU), Palem, India; ^18^Agricultural Research Station, Acharya NG Ranga Agricultural University (ANGRAU), Tirupati, India; ^19^Regional Agricultural Research Station, University of Agricultural Sciences (UAS), Vijayapura, India; ^20^Agricultural Research Station, Acharya NG Ranga Agricultural University (ANGRAU), Vizianagaram, India; ^21^International Crops Research Institute for the Semi-Arid Tropics (ICRISAT), Hyderabad, India

**Keywords:** representative, G × E, GGE-biplot, iron, zinc, grain yield, fodder yield

## Abstract

Pearl millet [*Pennisetum glaucum* (L.) R. Br.] is grown under both arid and semi-arid conditions in India, where other cereals are hard to grow. Pearl millet cultivars, hybrids, and OPVs (open pollinated varieties) are tested and released by the All India Coordinated Research Project on Pearl Millet (AICRP-PM) across three zones (A_1_, A, and B) that are classified based on rainfall pattern. Except in locations with extreme weather conditions, hybrids dominate pearl millet growing areas, which can be attributed to hybrid vigor and the active role of the private sector. The importance of OPVs cannot be ruled out, owing to wider adaptation, lower input cost, and timely seed availability to subsidiary farmers cultivating this crop. This study was conducted to scrutinize the presently used test locations for evaluation of pearl millet OPVs across India, identify the best OPVs across locations, and determine the variation in grain Fe and Zn contents across locations in these regions. Six varieties were evaluated across 20 locations in A_1_ and A (pooled as A) and B zones along with three common checks and additional three zonal adapted checks in the respective zones during the 2019 rainy season. Recorded data on yield and quality traits were analyzed using genotype main effects and genotype × environment interaction biplot method. The genotype × environment (G × E) interaction was found to be highly significant for all the grain yield and agronomic traits and for both micronutrients (iron and zinc). However, genotypic effect (G) was four (productive tillers) to 49 (grain Fe content) times that of G × E interaction effect for various traits across zones that show the flexibility of OPVs. Ananthapuramu is the ideal test site for selecting pearl millet cultivars effectively for adaptation across India, while Ananthapuramu, Perumallapalle, and Gurugram can also be used as initial testing locations. OPVs MP 599 and MP 600 are identified as ideal genotypes, because they showed higher grain and fodder yields and stability compared with other cultivars. Iron and zinc concentration showed highly significant positive correlation (across environment = 0.83; *p* < 0.01), indicating possibility of simultaneous effective selection for both traits. Three common checks were found to be significantly low yielders than the test entries or zonal checks in individual zones and across India, indicating the potential of genetic improvement through OPVs.

## Introduction

Pearl millet (*Pennisetum glaucum* L.R.Br.) is cultivated in dry regions of arid and semi-arid tropics where no other cereal can be successfully grown. India is the largest producer of millets in the world, harvesting about 11 million tons per year, nearly 36% of the world's output. Pearl millet, which accounts for about two-thirds of millet production in India, is grown in the drier areas of the country, mainly in the states of Rajasthan, Maharashtra, Gujarat, Uttar Pradesh, and Haryana. In India, pearl millet is the fourth most widely cultivated food crop after rice, wheat, and maize. It occupies an area of 6.93 million ha with an average production of 8.61 million tons and productivity of 1,243 kgha^−1^ (Directorate of Millets Development, 2020). The cultivated area for pearl millet in India is divided into three main zones based on soils and rainfall patterns. The northwestern part of India, receiving <400 mm of annual rainfall, is classified as an A_1_ zone. The northern and central parts of India, with sandy loam soils and receiving >400 mm of annual rainfall, are denoted as an A zone, and the peninsular region of India, receiving >400 mm of annual rainfall and bearing heavy soils, is broadly classified as a B zone (Rai et al., 2015). The arid tracts are grown with landraces/OPVs (open pollinated varieties) that are poor yielders. The progress achieved in pearl millet yields is attributed to the active role of the private sector in the dissemination of pearl millet hybrids in the productive zone of northern and central India rather than in the arid zone (Rai et al., 2015). On the other hand, the public sector could not record progress on par with that of the private sector. The active role of the private sector and the predominantly cross-pollinated nature of the crop have led to the rapid development and dissemination of hybrids pushing OPVs to marginal areas.

Pearl millet varieties have seed yields two to three times higher than inbred seed parents. The intra-population variability in pearl millet OPVs contributes toward greater resilience to several biotic and abiotic types of stress in contrast to the single-cross hybrids of pearl millet, which, when developed initially, were highly vulnerable to the downy mildew epidemic. The genetic heterogeneity of OPVs confers durable resistance to downy mildew, and a variation in flowering confers pollen-based escape from ergot and smut infection, which contrast well with frequent downy mildew epidemics and their greater vulnerability to ergot and smut. Consequently, improved pearl millet OPVs are readily acceptable to farmers and are easier to multiply, and hence have carved a niche for themselves even in India, where hybrids are the preferred cultivars (Sanjana Reddy, 2017). The genetic improvement of OPVs started in the 1930s and could not progress beyond a certain limit because of a narrow genetic base. During the 1970s, with the introgression of African germplasm lines especially from Western Africa, the primary center of diversity was led by the International Crops Research Institute for the Semi-Arid Tropics (ICRISAT) to enhance the genetic diversity of this crop. Due to such focused efforts, composites and OPVs were developed largely based on these germplasm lines. These populations were also a source for the breeding lines, which were widely used over the years by both the public and private sectors. The *Iniadi* germplasm, acquired from western Africa, has been extensively used in India, the USA, and other places worldwide, and several high-yielding hybrids were derived from it. OPV ICTP 8203 is also based on the *iniadi* germplasm. Apart from hybrids, OPVs such as WC-C75, Raj 171, ICMV 155, ICMV221, ICTP 8203, CZP 9802, and JBV 2 became very popular with farmers soon after their release (Yadav and Rai, 2013). In Maharashtra, a substantial proportion is still an OPV (variety ICTP 8203). In the arid tracts of west Rajasthan, landraces/OPVs are widely grown because of an extremely risky production environment. Though research focus has shifted to hybrids, the development of OPVs with good yield potential is possible. Hybrid parents with improved resistance to downy mildew and with good yield levels were further derived and formed the background of modern-day hybrids. These breeding lines were used worldwide, predominantly in India (Rai et al., 2014). However, the wide usage of a single source of germplasm, such as *iniadi*, poses a threat of disrupting existing heterotic patterns, which may be noticed in upcoming years. Diversification and development of new OPVs can also be used as a continuous source of variability for the generation of hybrid parental lines. Since OPVs are more preferred by resource-poor farmers in marginal areas, the nutritional status of OPVs with optimum levels of Fe and Zn has to be maintained, as the grain is consumed at the source. In this context, it becomes pertinent that the existing populations are evaluated for yield potential, adaptability, and nutritional status, and draw conclusive steps for the breeding and testing of new OPVS.

The research on Pearl millet improvement in India is carried out by the All India Coordinated Research Project on Pearl Millet (AICRP-PM), administered by the Indian Council of Agricultural Research (ICAR) through a network of 13 AICRP-PM centers and several voluntary ones. Newly developed cultivars are tested in multiple locations to determine their stability and performance before commercial release. Interpreting the genotype-by-environment interaction (GE) is essential for the identification of stable genotypes across environments to thereby obtain a correct ranking of genotypes and identify ideal genotypes for the target environment and ideal environment for discriminating genotypes. Several statistical methods are available for the study of GE. Among them, the additive main effects and multiplicative interaction (AMMI) and GGE biplots are frequently used for multi-environment trial (MET) data analysis. The GGE biplot analysis proposed by Yan et al. (2000) considers both genotype main effects and GEI effects for the analysis (Miranda et al., 2009), while genotype effects are not considered in AMMI. Therefore, the GGE biplot model is considered as an efficient method for identifying the best genotypes and test environments (Ding et al., 2007).

The current study was accomplished with the involvement of 20 locations across India to (i) identify best representative/ discriminating locations for the evaluation of pearl millet OPVs, (ii) identify stable OPVs across locations and those suitable to specific zones, and (iii) determine variation in grain Fe and Zn contents across locations in the OPVs.

## Materials and Methods

### Plant Material

The experimental material consisted of seven test or new varieties and nine released and popular OPVs, including biofortified variety Dhanshakti, used as checks in a population trial. Of them, seven test varieties and six checks were evaluated across 10 locations in zones A_1_ and A (pooled as A, referring to northern India), while six test varieties (new OPVs) and six checks were evaluated across 10 locations in zone B. For pooled analysis, six test varieties evaluated across 20 locations in the A and B zones, along with three common checks and additional three zonal-adapted checks in respective zones, during the 2019 rainy season were used. The information that pertains to the varieties used in this study is presented in Table 1.

**Table 1:** Varieties used in the study.

**Variety**	**Details**	**Testing zone**
**Test entries**		
MP 590 (VPMV 9)	Test variety bred at AICRP-PM, Vijayapura, Karnataka	A
MP 595 (GBL 2)	Test variety bred at AICRP-PAU, Ludhiana, Punjab	A, B
MP 596 (GBL 5)	Test variety bred at AICRP-PAU, Ludhiana, Punjab	A, B
MP 597 (HBC 53)	Test variety bred at AICRP-Hisar, Haryana	A, B
MP 598 (PC 720)	Test variety bred at ICAR-IARI, New Delhi	A, B
MP 599 (PC 721)	Test variety bred at ICAR-IARI, New Delhi	A, B
MP 600 (PC 722)	Test variety bred at ICAR-IARI, New Delhi	A, B
**Checks**		
PC 383	Bred at ICAR-IARI, New Delhi, released in 2001 for cultivation in A-zone	A
PC 701	Bred at ICAR-IARI, New Delhi, released in 2016 for cultivation in A-zone	A
JBV 2	Bred at AICPMIP Gwalior and ICRISAT, released in 1999 for cultivation in A-zone	A
ABV 04	Bred at ANGRAU, Ananthapuramu, released in 2019 for cultivation in B-zone	B
ICMV 155	Bred at ICRISAT, released in 1991 for cultivation in B-zone	B
PC 612	Bred at ICAR-IARI, New Delhi, released in 2011 for cultivation in B-zone	B
Raj 171	Bred at AICPMIP ARS Durgapura, Jaipur and ICRISAT, released in 1992 for cultivation across India	A, B
Dhanshakti (ICTP 8203 Fe)	Bred at ICRISAT, released in 2014 for cultivation across India	A, B
ICMV 221	Bred at ICRISAT, released in 1993 for cultivation across India	A, B

### Test Locations and Experiment

The multi-location testing was done at 20 locations in 11 states. The states of Maharashtra, Andhra Pradesh, and Rajasthan had three locations; the states of Karnataka, Haryana, and Madhya Pradesh were represented with two locations; and the states of Tamil Nadu, Telangana, Gujarat, Punjab, and Delhi were represented with a location each. Detailed features of these test locations and dates of sowing are given in Table 2. The crops were sown with the onset of monsoon in each of these locations. In each location, the experiment was conducted in a randomized complete block design with three replications. The plot size of each genotype varied from 12 to 14.4 m^2^ across locations, which included approximately 100 plants. Plot yield data were converted to tons per hectare using the plot size as a factor.

**Table 2:** Test locations used in the study.

**Location**	**Soil**	**pH**	**Date of sowing**	**Latitude**	**Longitude**	**Altitude**
**Zone A**						
Mandor	Sandy loam	8.1	22 July	26°34′ N	73°05′ E	241 m
Bikaner	Sandy	8.0	25 July	28°01′ N	73°30′ E	250 m
Durgapura	Sandy loam	–	8 July	26°90′ N	75°80′ E	425 m
Jamnagar	Medium black	7.6	2 August	22°28′ N	70°05′ E	17 m
Hisar	Sandy loam	–	27 June	29°15′ N	75°70′ E	215 m
Gurugram	–	–	5 July	28°46′ N	77°03′ E	217 m
Gwalior	Sandy loam	7.5	13 July	26°22′ N	78°18′ E	211 m
Morena	Clay loam	7.3	22 June	26°49′ N	77°99′ E	177 m
Ludhiana	Sandy loam	8.0	18 July	30°90′ N	75°85′ E	262 m
New Delhi	Sandy loam	7.8	10 July	28°61′ N	77°20′ E	216 m
**Zone B**						
Aurangabad	Medium black	7.5	2 July	19°86′ N	75°30′ E	568 m
Niphad	Medium black	8.8	13 July	20°08′ N	74°10′ E	569 m
Dhule	Medium black	8.6	7 July	21°08′ N	74°01′ E	210 m
Vijayapura	Shallow black	8.7	21 July	16°50′ N	75°43′ E	594 m
Malnoor	Medium black	8.2	20 July	17°03′ N	76°15′ E	460 m
Ananthapuramu	Red sandy loam	6.5	9 August	14°68′ N	77°60′ E	335 m
Perumallapalle	Red sandy	6.9	15 July	13°39′ N	79°35′ E	183 m
Vizianagaram	Red sandy loam	6.4	15 July	18°10′ N	83°39′ E	74 m
Palem	Sandy loam	7.9	27 June	16°35′ N	78°10′ E	642 m
Coimbatore	Clay loam	7.8	2 July	11°02′ N	76°96′ E	427 m

### Trait Measurements

Eight yield-related traits were measured in each trial. The flowering time (DF) was measured as the number of days taken from the date of sowing to the date on which 50% of plants in a plot showed full stigma emergence. The length in centimeters of fully matured plants from the base of the plant to the top of the ear head was recorded as plant height (PHT). The number of productive tillers (NPT) was counted as the total number of tillers that bear ear head with grains per plant. The panicle length (PL) was measured from the base of the panicle to its tip and recorded in centimeters. The panicle diameter (PD) was measured at the maximum thickness of the panicle in centimeters. Grain yield (GY) was estimated by weighing the grains obtained after drying and threshing of the panicles at 12% moisture content and expressed in grams. Then, the weight per plot was extrapolated into t/ha. For measuring dry fodder yield (DFY), harvested plants were allowed to sun-dry for 7–10 days. The weight recorded per plot was extrapolated to t/ha. For measuring 1,000-grain weight (1,000 GWT), a sample of 1,000 grains was counted randomly from the threshed seed, and the weight was recorded in grams.

### Micronutrient Analysis

The grain Fe and Zn contents were analyzed using an energy-dispersive x-ray fluorescence spectrometry machine (ED-XRF), model X-Supreme 8000 from OXFORD, installed in the Pearl Millet Breeding program at ICRISAT, Patancheru, India. The ED-XRF method for pearl millet established and reported a higher correlation between ICP-OES and ED-XRF (*r* = 0.9) for both Fe and Zn, as suggested by Govindaraj et al. (2016). The quantified grain iron and zinc levels were measured in milligrams per kilogram (mg kg^−1^) of the seed and interpreted in the same unit. All possible care was taken from sampling to laboratory analysis to avoid any contamination.

### Statistical Analysis

Recorded data from eight yields and two quality traits were subjected to combined analysis of variance (ANOVA) to investigate genotypes (G), environments (E) and genotype × environment interaction (GEI) effects using GenStat 12th edition. In the combined ANOVA, the genotypes were considered as fixed effects, while the environments and replications were considered as random effects. As the GEI was significant, GGE biplot method (Yan et al., 2000) was employed to analyze GE interaction and assess the stability of GY, DFY, Fe and Zn data, and the pattern of response of OPVs tested in 20 locations. For the eight individual trials, the Pearson's correlation coefficients were calculated using R-software (R Development Core Team, 2019). The repeatability of a variety trial is derived as the proportion of the variation due to genotype effects. Variance components and heritability across the locations were estimated. The broad-sense heritability was calculated as: *h*^2^ = ${б}_{\text{g}}^{2}$/(${б}_{\text{g}}^{2}$ + ${б}_{\text{gl}}^{2}$/l + б2_e_/lr), where ${б}_{\text{g}}^{2}$ is the genotypic variance, ${б}_{\text{gl}}^{2}$ is the interaction variance of genotype with location, ${б}_{\text{e}}^{2}$ was the error variance, l was the number of locations, and r was the number of replicates. The estimates of б^2^g, ${б}_{\text{gl}}^{2}$, ${б}_{\text{e}}^{2}$ were obtained from an ANOVA with the environment considered as a random effect, as mentioned by Xie et al. (2020).

The grain yield stability of OPVs and suitability of test environments is tested using the GGE biplot based on the following model proposed by Santos et al. (2019).

$$\begin{array}{l} {\text{Y}}_{\text{ij}}-{\text{Y}}_{\text{j}}={\text{λ}}_{1}{\text{ξ}}_{\text{i}1}{\text{η}}_{1\text{j}}+{\text{λ}}_{2}{\text{ξ}}_{\text{i}2}{\text{η}}_{2\text{j}}+\;{\text{ε}}_{\text{ij}} \end{array}$$

where Y_ij_ is the mean grain yield of genotype i in environment j; Yj is the mean grain yield of environment j; λ_1_ and λ2 are the singular values of the first and second principal components, PC1 and PC2, respectively; ξ_i1_ and ξ_i2_ are the scores of genotype i for PC1 and PC2, respectively; η_1j_ and η_2j_ are the scores of environment j for PC1 and PC2, respectively; and εij is the error associated with the model.

The genotype-centered and the environment-centered singular value partitioning (SVP) are used for the evaluation of genotypes and environments, respectively (Yan et al., 2011), but symmetric scaling is preferred for the study of which-won-where pattern (Yan, 2002). Genotype-by-trait biplot (GT biplot) is generated from combined data using “Genotype-by-trait biplots Scaling = 1 option” of GGE biplot software. Here, traits were considered as “tester.” “Which is best for what” analysis is performed to identify the genotypes superior for particular traits. GGE Biplot analyses were performed using the R statistical software, version 4.0.0 (R Development Core Team, 2019) and GGEbiplot ver. 8.2 (Yan, 2001).

## Results

### Analysis of Variance

ANOVA was performed zone-wise as well as pooled over all the locations. The combined ANOVA across environments evidenced highly significant differences among genotypes for all the recorded traits. The proportion of genotype to GE variance was 4–5 times for NPT and DFY; 9–10 times for GY, PL, and 1,000 GWT; 12–15 times for DF, PHT, PW, and Zn; and 49 times for Fe. The proportion of genotypic variance to total variance was marginally higher for traits GY and 1,000 GWT in the A-zone in contrast to the B-zone; while for other traits, the genotypic variance was marginally superior in the B-zone. The broad-sense heritability estimates for NPT (0.73) and DFY (0.79) were lower than those of the other traits (0.88–0.98). The repeatability of the trial, as measured by broad-sense heritability, was marginally higher for traits GY and DFY in the A-zone; while for the other traits, B-zone estimates were marginally higher (Table 3).

**Table 3:** Estimation of variance components and broad sense heritability (h^2^bs) for yield, quality, and agronomic traits in A-zone, B-zone, and across all locations in India.

**Statistics**	**GY**	**DFY**	**DF**	**PHT**	**NPT**	**PL**	**PD**	**1,000 GWT**	**Fe**	**Zn**
**A-zone**										
h^2^bs	0.79	0.68	0.84	0.9	0.5	0.83	0.82	0.75	0.93	0.83
Genotype variance	2.1	36.7	112.4	6517.2	1.0	118.6	0.9	8.0	2053.3	345.1
Location variance	8.8	1244.3	633.0	43850.9	15.0	389.6	3.6	31.2	2913.9	3331.6
Genotype × location variance	0.4	11.9	17.8	639.0	0.5	19.6	0.2	2.0	134.4	58.1
Residual variance	0.1	1.4	1.9	165.6	0.1	2.7	0.0	0.3	83.8	30.4
LSD (*P* < 0.05)	0.4	1.9	2.2	20.7	0.5	2.7	0.3	0.8	14.7	8.9
CV (%)	11.1	14.5	2.8	6.1	14.2	6.0	7.5	5.5	16.8	14.9
**B-zone**										
h^2^bs	0.74	0.62	0.9	0.91	0.71	0.89	0.91	0.82	0.97	0.86
Genotype variance	1.9	13.8	144.5	5294.2	2.0	92.6	1.6	28.2	2507.4	336.0
Location variance	28.2	120.0	289.9	32110.1	13.5	169.3	1.8	153.7	3126.9	1588.7
Genotype × location variance	0.5	5.3	14.5	476.8	0.6	10.6	0.1	5.0	69.9	46.0
Residual variance	0.1	0.6	2.2	104.0	0.2	3.1	0.0	0.7	19.1	16.1
LSD (*P* < 0.05)	0.5	1.2	2.4	16.4	0.8	2.8	0.3	1.3	7.0	6.5
CV (%)	15.9	14.7	3.1	5.5	18.5	7.2	7.7	6.7	9.4	12.9
**Pooled**										
h^2^bs	0.88	0.79	0.93	0.94	0.73	0.9	0.92	0.89	0.98	0.93
Genotype variance	4.1	41.5	326.5	10873.7	1.8	198.9	2.1	33.7	5651.7	816.1
Location variance	13.0	485.0	682.3	28085.6	9.4	272.4	2.1	116.4	2727.3	1992.9
Genotype × location variance	0.5	8.5	23.1	704.4	0.5	19.3	0.2	3.6	114.8	53.2
Residual variance	0.1	0.8	2.1	146.1	0.1	3.3	0.0	0.5	56.7	24.4
LSD (*P* < 0.05)	0.4	1.5	2.4	19.4	0.6	2.9	0.3	0.4	12.1	7.9
CV (%)	13.6	14.3	3.0	6.2	16.0	7.1	7.2	13.6	14.6	14.2

### Mean Performance

The OPVs had better performance for DFY, PHT, PL, Fe, and Zn in the A-zone; GY, NPT, and 1,000 GWT in the B-zone, while no difference was observed for DF and PD across zones. The new OPVs had 27% higher grain yield in the A zone and 25% higher in the B zone compared with the checks. In both zones, varieties MP 596, MP 599, and MP 600 had the highest grain yield (2.18–2.35 t/ha). Similarly, for DFY, new varieties were superior to checks by 27% in the A-zone and 23% the in B-zone. OPVs MP 595, MP 599, and MP 600 had a higher DFY of 8.74–9.42 t/ha in the A zone; while OPVs MP 598, MP 599, and MP 600 had a higher DFY of 5.6–5.96 t/ha in the B zone. Thus, the two OPVs, MP 599 and MP 600, were superior for both GY and DFY. The biofortified variety, Dhanshakti, had the highest levels of Fe (72.1 ppm) and Zn (41.4 ppm), followed by check ICMV 221. The new OPVs had lesser levels of Fe (21% in A-zone, 25% in B-zone), and Zn (14% in A-zone, 16% in B-zone) compared with checks. Among the new OPVs, MP 597 had higher levels of Fe (53.1 ppm) and Zn (35.5 ppm). When the other agronomic traits were observed across the zones, the new OPVs were late by 3 days, had a taller height of 7–15 cm, longer panicle length of 2.5 cm, and smaller grain size by 0.2–0.5 g/1,000 grains. Minor differences were observed for PD and NPT (Table 4).

**Table 4:** Performance of OPVs for grain yield and quality traits pooled across locations.

**Varieties/checks**		**GY**	**DFY**	**DF**	**PHT**	**NPT**	**PL**	**PD**	**1,000 GWT**	**Fe**	**Zn**
MP 595	A	2.08	9.42	51.0	241.9	2.7	29.7	2.4	8.3	49.4	38.3
	B	1.96	4.38	48.3	201.1	2.7	25.4	2.2	10.8	44.0	34.0
	AB	2.02	6.90	50.4	221.2	2.6	27.5	2.3	9.5	47.1	36.4
MP 596	A	2.31	8.23	50.9	197.7	2.1	26.6	2.9	9.6	51.7	32.3
	B	2.35	5.09	49.5	176.3	2.6	23.2	2.8	12.5	45.0	29.0
	AB	2.33	6.66	50.5	185.0	2.3	25.0	2.7	11.0	48.4	30.7
MP 597	A	1.73	6.89	48.6	196.7	2.5	27.7	2.8	9.3	59.5	38.6
	B	1.97	4.67	50.0	160.1	2.6	25.0	2.8	13.1	46.0	32.0
	AB	1.85	5.78	49.9	182.6	2.5	26.0	2.8	11.1	53.1	35.5
MP 598	A	2.06	8.04	47.7	212.3	2.2	28.0	2.6	9.1	46.8	33.1
	B	2.17	5.60	47.6	186.5	2.4	25.3	2.7	12.4	41.0	28.0
	AB	2.12	6.82	48.2	201.2	2.3	27.0	2.6	10.7	44.2	30.7
MP 599	A	2.18	9.01	49.8	208.8	2.2	26.8	2.8	9.3	49.9	35.8
	B	2.34	5.82	48.7	195.8	2.4	25.5	2.7	12.0	39.0	29.0
	AB	2.26	7.41	49.4	201.8	2.3	25.9	2.8	10.6	45.0	32.5
MP 600	A	2.25	8.74	48.5	215.6	2.3	28.9	2.7	9.2	49.0	35.7
	B	2.22	5.96	48.0	197.0	2.4	24.7	2.6	12.1	41.0	28.0
	AB	2.24	7.35	48.9	207.2	2.4	27.0	2.7	10.6	45.1	31.9
Mean of new OPVs	**A**	**2.10**	**8.39**	**49.4**	**212.2**	**2.3**	**28.0**	**2.7**	**9.1**	**51.1**	**35.6**
	**B**	**2.17**	**5.25**	**48.7**	**186.1**	**2.5**	**24.9**	**2.6**	**12.2**	**42.7**	**30.0**
	**AB**	**2.14**	**6.82**	**49.5**	**199.8**	**2.4**	**26.4**	**2.7**	**10.6**	**47.2**	**33.0**
Raj 171	A	1.69	6.02	49.2	208.0	2.5	28.4	2.4	8.7	51.9	39.7
	B	1.75	4.28	49.4	191.1	3.0	24.7	2.3	10.6	42.0	34.0
	AB	1.72	5.15	49.9	200.9	2.6	26.7	2.4	9.6	47.1	36.8
Dhanshakti	A	1.58	7.35	45.8	186.9	2.0	25.4	2.8	10.2	74.3	44.3
	B	1.69	4.91	42.7	170.7	2.4	20.7	2.9	13.6	70.0	38.0
	AB	1.63	6.13	44.4	180.8	2.2	23.3	2.8	11.8	72.1	41.4
ICMV 221	A	1.69	6.47	44.2	194.8	2.1	22.6	2.8	9.8	67.3	40.8
	B	1.78	4.23	44.6	175.7	2.3	21.8	2.9	12.9	59.0	35.0
	AB	1.74	5.35	44.7	187.5	2.2	22.2	2.8	11.3	63.4	38.0
Mean of checks	**A**	**1.65**	**6.61**	**46.4**	**196.6**	**2.2**	**25.5**	**2.7**	**9.6**	**64.5**	**41.6**
	**B**	**1.74**	**4.47**	**45.6**	**179.2**	**2.6**	**22.4**	**2.7**	**12.4**	**57.0**	**35.7**
	**AB**	**1.70**	**5.54**	**46.3**	**189.7**	**2.3**	**24.1**	**2.7**	**10.9**	**60.9**	**38.7**
Trial mean		1.99	6.39	48.5	196.5	2.4	25.6	2.7	10.7	51.7	34.9
Lsd (5%) b/w entries		0.10	0.33	0.5	4.3	0.1	0.7	0.1	0.2	2.7	1.8

*GY, grain yield (t/ha); DFY, dry fodder yield (t/ha); DF, days to 50% flowering; PHT, plant height (cm); NPT, number of productive tillers; PL, panicle length (cm); PD, panicle width (cm); 1,000 GWT, 1,000 grain weight (g); Fe, Fe (mg/kg); Zn, Zn (mg/kg). Mean values are in bold font*.

Among the A-zone locations, Ludhiana, Gurugram, and Hisar were more productive for GY (2.3–2.6 t ha^−1^) while Gurugram, Ludhiana, and New Delhi were productive for DFY (12.1–17.4 t ha^−1^). The grains harvested from New Delhi, Hisar, and Bikaner had higher levels of Fe and Zn contents. In the B-zone, the Aurangabad, Ananthapuramu, and Vizianagaram locations had higher grain yields (2.7–3.2 t ha^−1^), while the Perumallapalle, Vijayapura, and Malnoor locations had higher fodder yields (5.9–9.3 t ha^−1^). The grains harvested from the Dhule, Aurangabad, and Malnoor locations had higher levels of Fe and Zn contents (Table 5).

**Table 5:** Location and zonal means for grain yield and quality traits pooled across OPVs.

**Location**	**GY**	**DFY**	**DF**	**PHT**	**NPT**	**PL**	**PD**	**1,000 GWT**	**Fe**	**Zn**
Bikaner	1.69	6.78	46.8	168.9	2.0	24.1	2.6	8.9	61.9	42.6
Durgapura	1.93	2.49	48.7	171.5	2.1	25.8	2.3	9.1	51.8	34.7
Gurugram	2.47	17.36	49.9	248.2	1.0	34.8	3.0	8.3	53.9	38.9
Gwalior	2.01	7.86	47.9	224.5	2.4	25.5	2.3	9.2	53.5	19.9
Hisar	2.30	6.33	53.4	257.4	2.9	29.0	2.9	9.3	61.3	48.3
Jamnagar	0.96	2.75	43.6	177.6	1.8	25.0	2.6	8.1	52.7	33.4
Ludhiana	2.62	12.07	51.9	212.7	2.7	29.5	3.2	10.0	40.4	33.2
Mandor	1.60	2.08	48.7	171.5	2.1	25.8	2.3	9.1	50.3	34.6
Morena	1.81	4.72	40.6	224.1	3.3	26.4	2.8	9.5	–	–
New Delhi	2.13	15.52	52.7	213.3	2.6	25.4	2.8	11.5	74.1	53.1
A-zone mean	1.95	7.80	48.42	207.0	2.3	27.1	2.7	9.3	55.2	37.4
Ananthapuramu	2.82	3.85	45.4	178.7	2.9	25.2	2.5	14.9	47.2	25.8
Aurangabad	3.15	4.66	51.3	173.7	1.9	23.0	2.7	13.0	52.0	37.0
Coimbatore	2.31	4.77	46.0	213.2	3.3	23.9	2.5	11.0	31.1	40.0
Dhule	2.23	5.00	51.3	224.2	2.5	26.4	3.1	10.2	64.5	39.2
Malnoor	1.81	5.89	48.3	179.3	3.3	24.3	2.7	11.7	51.4	28.4
Niphad	0.13	3.61	63.3	165.7	2.3	21.0	2.3	10.7	–	–
Palem	1.12	2.15	43.7	161.6	2.6	20.2	2.3	10.6	43.0	22.7
Perumallapalle	1.85	9.29	48.3	204.3	1.6	26.7	2.7	11.8	42.9	29.4
Vijayapura	2.10	6.55	46.7	135.2	2.0	22.6	2.8	11.3	47.3	31.7
Vizianagaram	2.74	4.15	40.9	224.0	2.2	27.7	2.9	15.6	–	–
B-zone mean	2.03	4.99	48.5	186.0	2.5	24.1	2.7	12.1	48.3	32.4
lsd (*P* < 0.05) across locations	0.14	0.49	0.8	6.5	0.2	1.0	0.1	0.4	4.0	2.6

*GY, grain yield (t/ha); DFY, dry fodder yield (t/ha); DF, days to 50% flowering; PHT, plant height (cm); NPT, number of productive tillers; PL, panicle length (cm); PD, panicle width (cm); 1,000 GWT, 1,000 grain weight (g); Fe, Fe (mg/kg); Zn, Zn (mg/kg)*.

### Trait Associations

**Figure 1:**
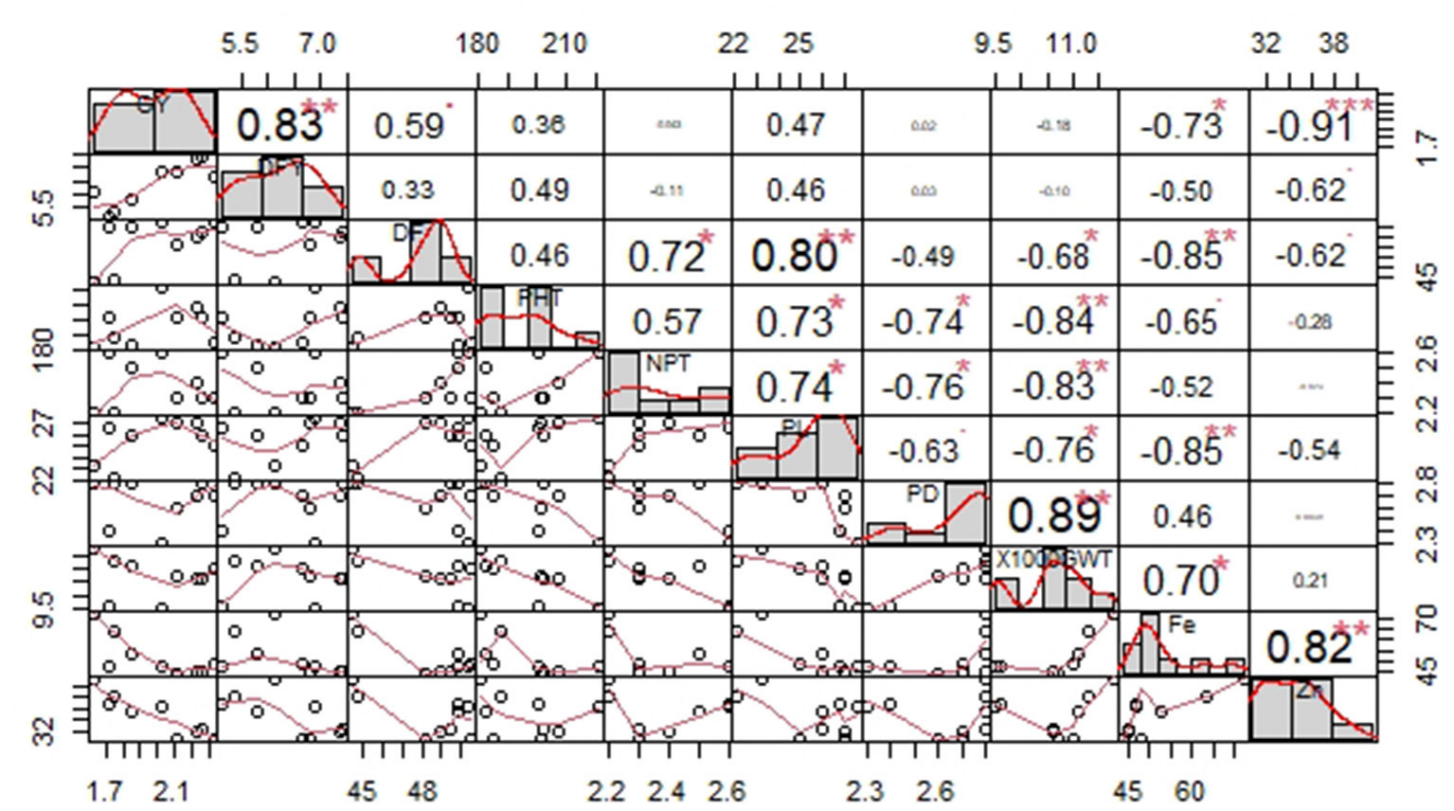
Correlation among yield, quality, and agronomic traits recorded on nine OPVs over 20 locations. Significant effects at *P < 0.05, **P < 0.01, ***P < 0.001.

Increase in grain yield was significantly associated with the enhancement of DFY. However, the grain Fe and Zn contents decreased with an increase in grain yield. Grain Fe content was more in early flowering OPVs with shorter panicles and bigger seed sizes. High Fe content is significantly related to high Zn content in the grain (Figure 1).

### Mean Performance and Stability Visualized Through Genotype Main Effect Plus Genotype by Interaction Biplot

**Figure 2:**
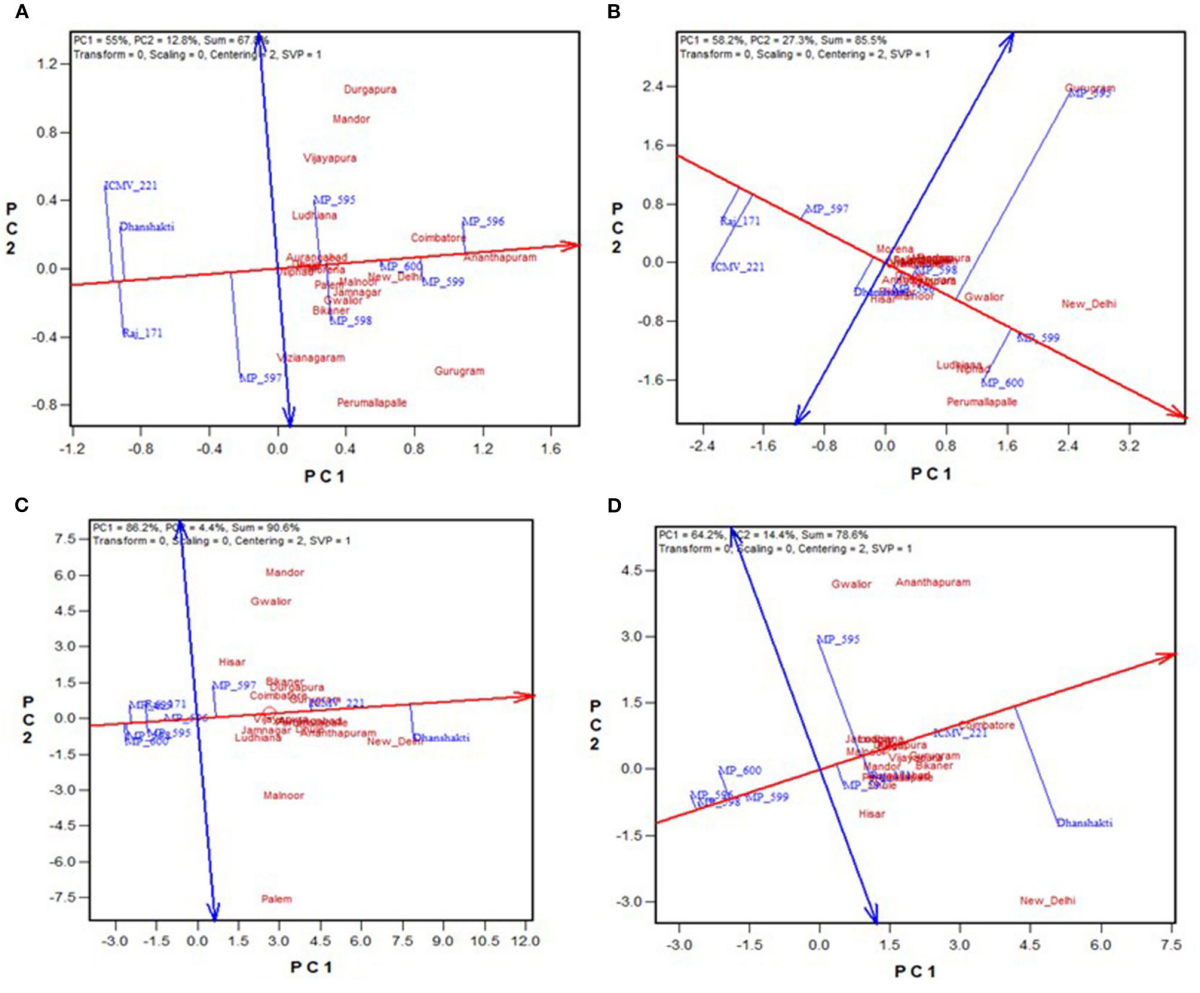
GGE biplot showing “mean vs. stability” of nine pearl millet OPVs across 20 locations for **(A)** grain yield, **(B)** dry fodder yield, **(C)** grain Fe content, and **(D)** grain Zn content.

The environment-centered (centering = 2) genotype-metric (SVP = 1) biplots without scaling (scaling = 0) for grain yield and fodder yield, economically important traits and Fe and Zn, and grain quality traits are presented in Figures 2A–D, respectively. The first two PCs explained the 67.8% variation for GY, 85.5% for DFY, 90.4% for Fe, and 78.7% for Zn. The AEC abscissa passes through the biplot origin and acts as a marker for the average environment and points toward higher mean values (Yan, 2001). The perpendicular lines to the AEC passing through the biplot origin are referred to as AEC ordinate. These ordinates are depicted as dotted lines in Figures 2A–D. The greater the absolute length of the projection of a cultivar, the less stable it is. Furthermore, the average yield of genotypes is approximated by the projections of their markers to the AEC abscissa (Kaya et al., 2006). Accordingly, MP 596 was the best performing genotype in terms of grain yield, followed by MP 599 and MP 600; while ICMV 221, Raj 171, and Dhanshakti were limited by lower yields. They were also least stable for grain yield with higher projection from the AEC abscissa. OPV MP 600 was the most stable among the high-yielding OPVs (Figure 2A). For dry fodder yield, MP 599 and MP 600 had the highest yields, but MP 599 was more stable. Compared to the test varieties, the check varieties had a low fodder yield (Figure 2B). Dhanshakti had higher Fe (72.1 mg/ka) and Zn (41.4 mgkg^−1^) contents, followed by ICMV 221 (63.4 mgkg^−1^ Fe and 38 mgkg^−1^ Zn). However, ICMV 221 had greater stability for the traits. The rest of the OPVs did not perform well for grain quality traits (Figures 2C,D).

### Relationship Among Environments

**Figure 3:**
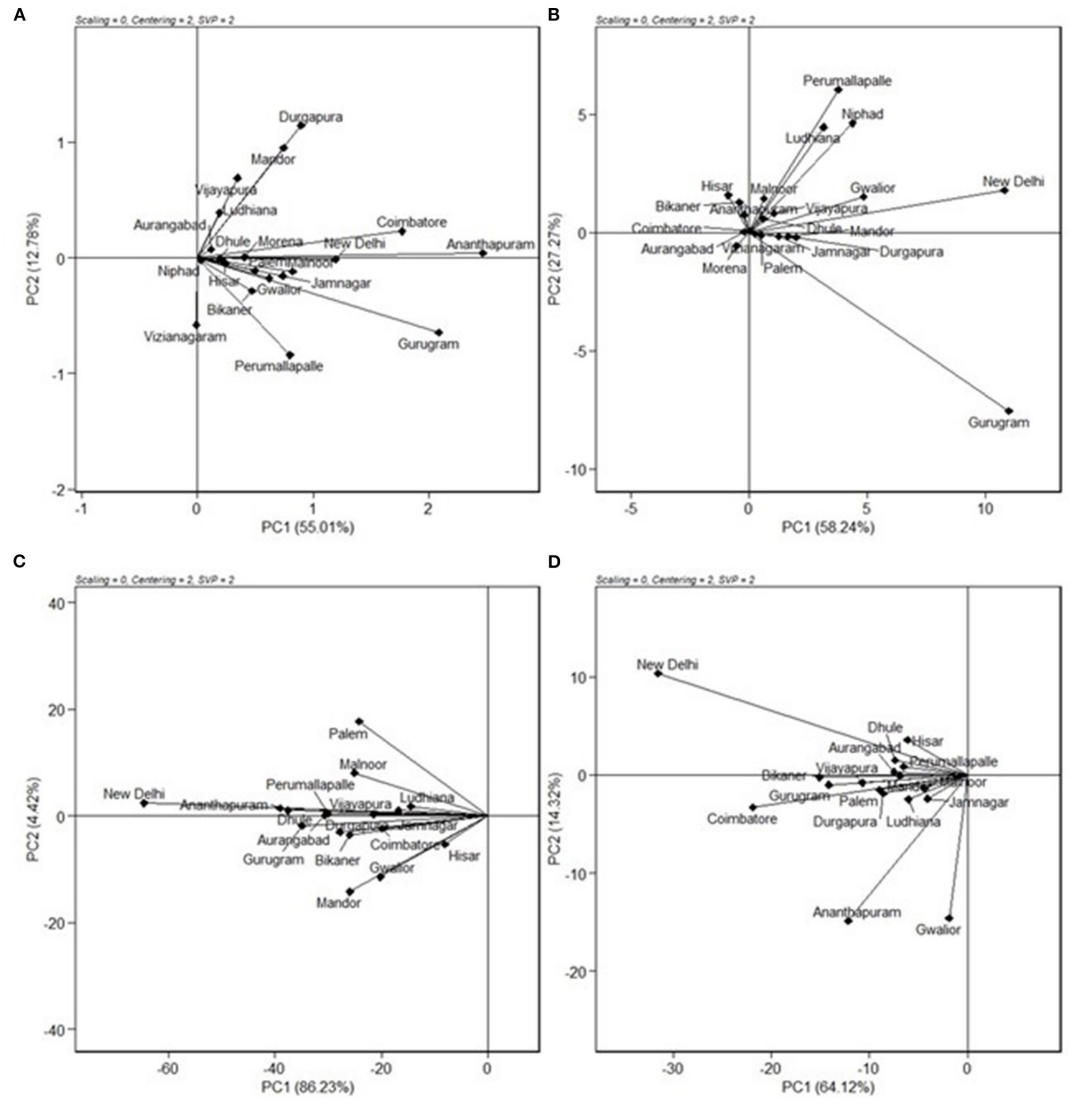
GGE biplot showing “relationship among environments” of nine pearl millet OPVs across 20 locations for **(A)** grain yield, **(B)** dry fodder yield, **(C)** grain Fe content, and **(D)** grain Zn content.

The relationships among the test environments were studied by environment centered (centering = 2), environment metric (SPV = 2), and without scaling (scaling = 0). Combined ANOVA for grain yield (Figure 3A) showed that the majority of the angles between their vectors are acute. Acute vector angles are indicative of a closer relationship among the environments (Yan and Tinker, 2006). Thus, the majority of the locations were highly correlated except for the Vizianagaram and Perumallapalle locations and for the Ludhiana, Vijayapura, Mandor, and Durgapura locations, which shows no relationship among them as the angle was 90°. The distance between two environments measures their ability to discriminate genotypes. Thus, the 20 locations could be divided into three groups for grain yield; one with Durgapura, Mandor, Vijayapura, and Ludhiana; second with Vizianagaram and Perumallapalle; and the other 14 locations forming the third group. The groupings did not correlate with the A-zone and B-zone groupings that exist or with geographical identity. The environments were diverse with respect to fodder yield. The locations Perumallapalle, Niphad, Ludhiana, Malnoor, Gwalior, Vijayapura, New Delhi, Dhule, Mandor, and Durgapura were related for DFY (Figure 3B). All of the locations were highly correlated for grain Fe content (Figure 3C), while the majority of the locations were correlated for grain Zn content (Figure 3D) with the exception between Gwalior and Hisar, which has a right angle between them showing no-relationship.

**Figure 4:**
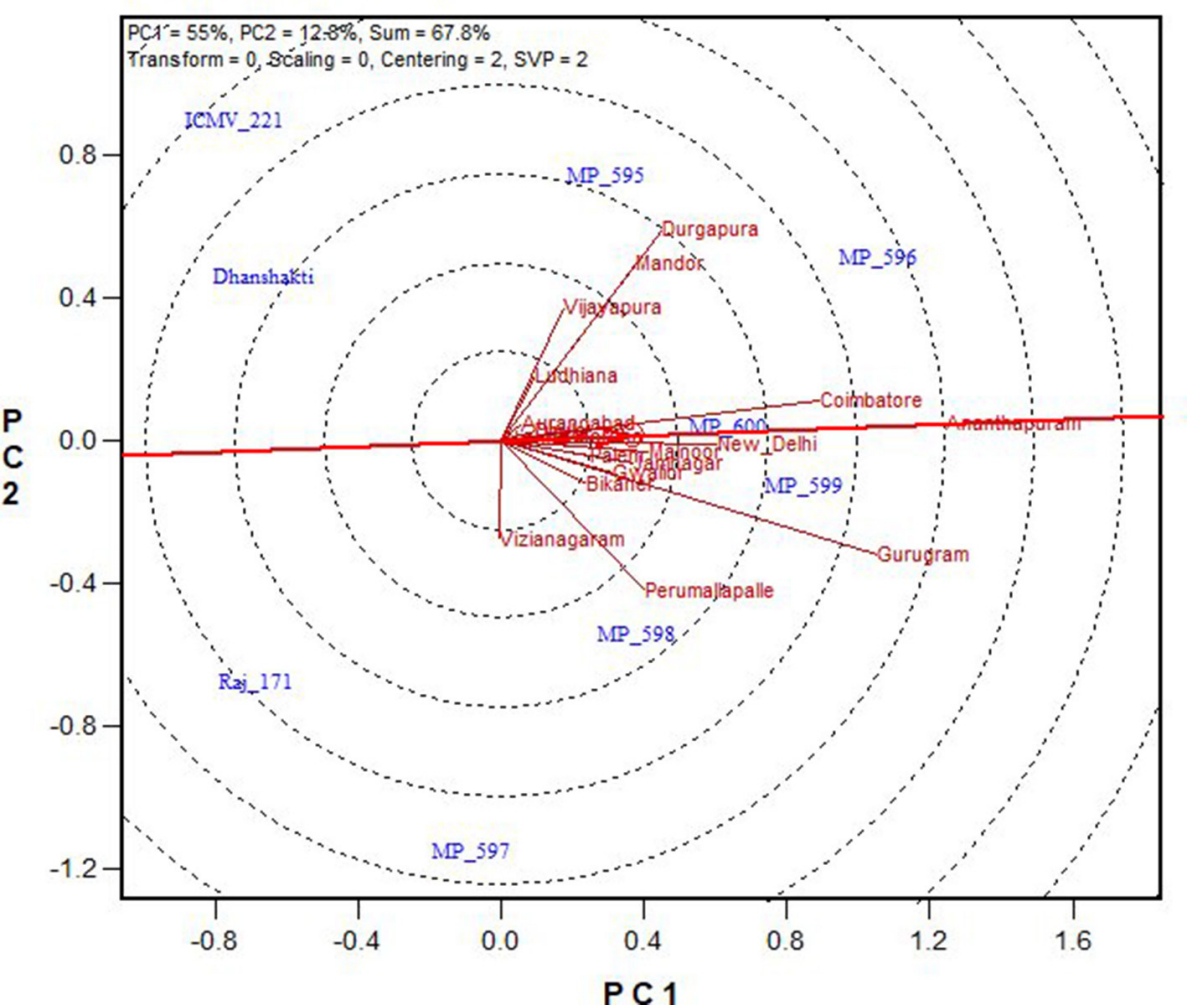
Ranking of environments for grain yield based on discriminating ability and representativeness.

**Figure 5:**
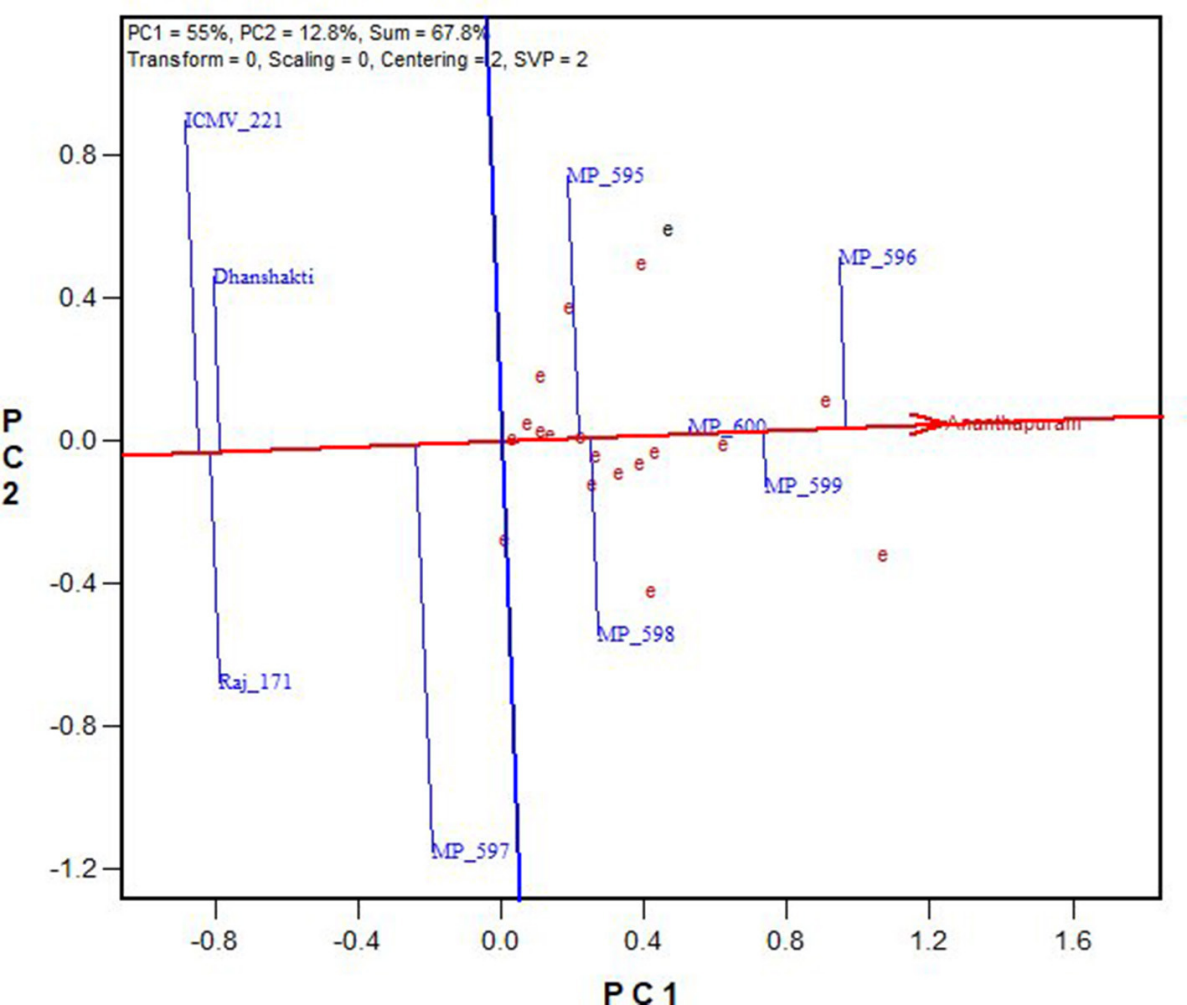
Ranking of genotypes based on their performance for grain yield in near-ideal location, Ananthapuramu.

In Figure 4, “average environment” is represented by a small circle on the average environment axes (AEA). The length of environmental vectors is proportional to the standard deviation of the genotypes in the environments. The longer environmental vectors indicate that the environment is more differentiating for the trait among the genotypes. Another important criterion in evaluating environments is the test of their representativeness. The average environment coordination (AEC) line crosses the center of the biplot and the medium environment, and the angle of each vector with the AEC axis is a criterion for identifying the sample environment. Environments with smaller angles with the AEA are most representative of the average test environments. A suitable environment should have two criteria at the same time: distinctive and a target environment. The Ananthapuramu location was closest to the average environment, and thus is the most representative or discriminating environment, followed by the New Delhi location. While ranking the genotypes in near-average environment Ananthapuramu, MP 596, MP 599, and MP 600 had higher GY; MP 595 and MP 598 had moderate yield, and genotypes MP 597, Raj171, ICMV 221, and Dhanshakti had lower than average yield. Variety MP 600 was highly stable, followed by MP 599 (Figure 5).

### Which Won Where and Mega Environment Identification

**Figure 6:**
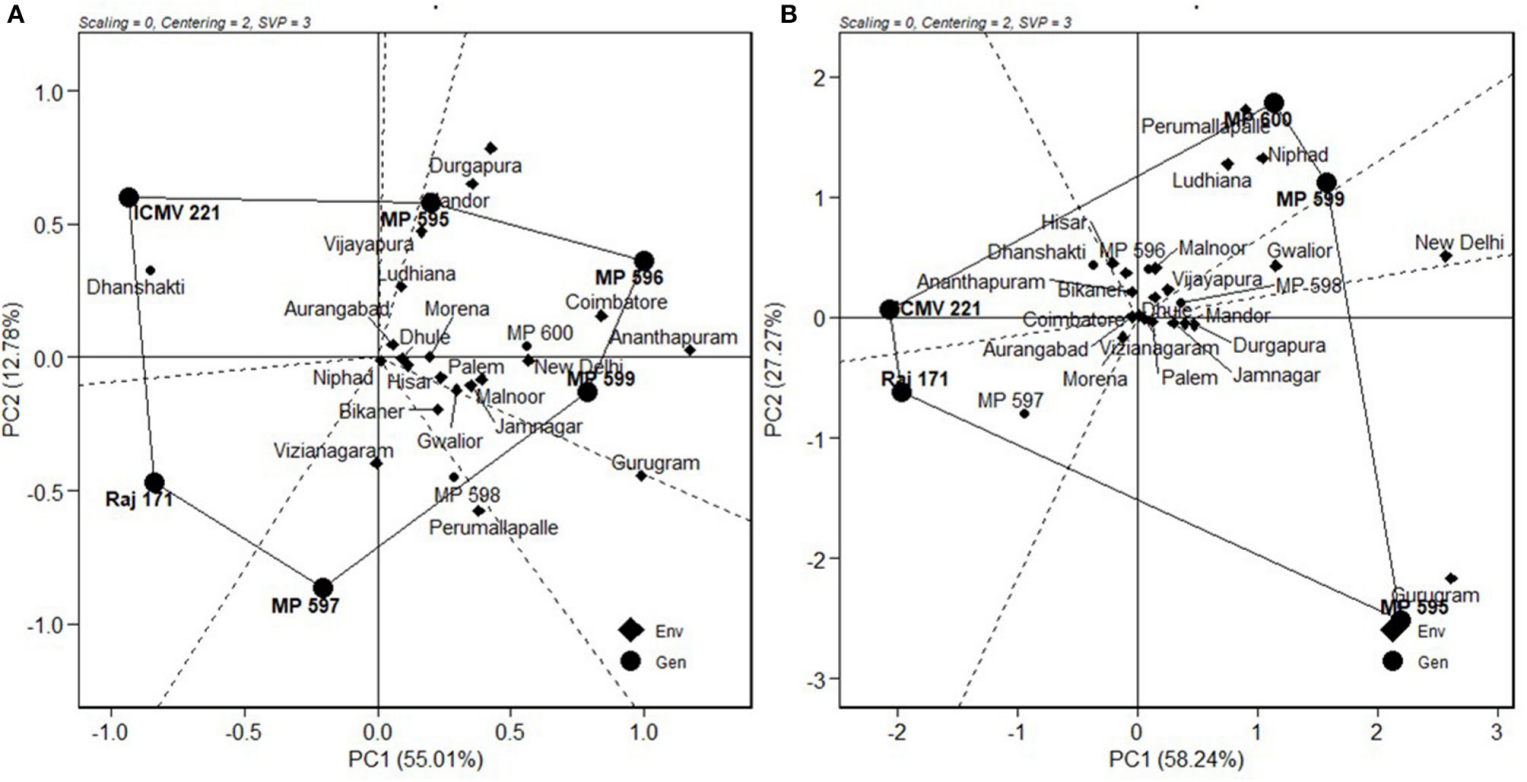
Which-won-where analysis of the genotypes for **(A)** grain yield and **(B)** fodder yield.

A mixture of crossover and non-crossover types of GEI in MET data is of very common occurrence (Kaya et al., 2006; Fan et al., 2007; Sabaghnia et al., 2008; Rao et al., 2011). The “which-won-where feature” of the GGE biplot graphically addresses crossover GE, mega-environment differentiation, specific adaptation, etc. (Gauch and Zobel, 1997; Yan et al., 2000; Yan and Tinker, 2006; Putto et al., 2008; Rao et al., 2011). The “which-won-where” graph is constructed by joining the farthest genotypes in a polygon. From the origin of the biplot, perpendicular lines, referred to as equity lines, are drawn to the sides of the polygon, separating the polygon into several sectors (Yan, 2001). Genotype at the vertex is the best performing genotype in the environment falling in that sector (Yan and Tinker, 2006). The “which-won-where” biplots for GY and DFY over pooled locations are presented in Figures 6A,B. The biplots indicated the existence of crossover GEI and the existence of mega-environments (ME). For grain yield over pooled locations, the hexagon has six genotypes, MP 595, MP 596, MP 599, MP 597, Raj 171, and ICMV 221 at its vertices. The equity lines divided the biplot into six sectors, of which three retained 20 locations. The testing locations partitioned into three MEs, ME1 with locations Vijayapura, Ludhiana, Dhule, Aurangabad, Morena, Palem, Jamnagar, Malnoor, Hisar, Jamnagar, New Delhi, Ananthapuramu, Mandor, Durgapura, and Coimbatore with MP 595, MP 596, and MP 599 as the winning genotypes. ME2 consisted of locations Vijayanagaram, Perumallapalle, and Niphad with MP 597 as the winning genotype. ME3 consisted of the Bikaner and Gurugram locations with no genotype performing better for these locations (Figure 6A). The correlation among the locations did not exist in terms of geography. For fodder yield, five genotypes were placed at the vertices of the pentagon, and the biplot was divided into five sectors. ME1 had seven locations: Perumallapalle, Niphad, Ludhiana, Malnoor, Vijayapura, Hisar, Bikaner, and Ananthapuramu with MP 599 and MP 600 as winning genotypes. The second largest ME had six locations: Mandor, Durgapura, Jamnagar, Palem, Gurugram, and Dhule with MP 595 as the winning genotype. ME3 had Coimbatore, Aurangabad, Morena, and Vijayanagaram with Raj171 as the winning genotype; while Gwalior and New Delhi fell into ME4, which had no winning genotype (Figure 6B).

**Figure 7:**
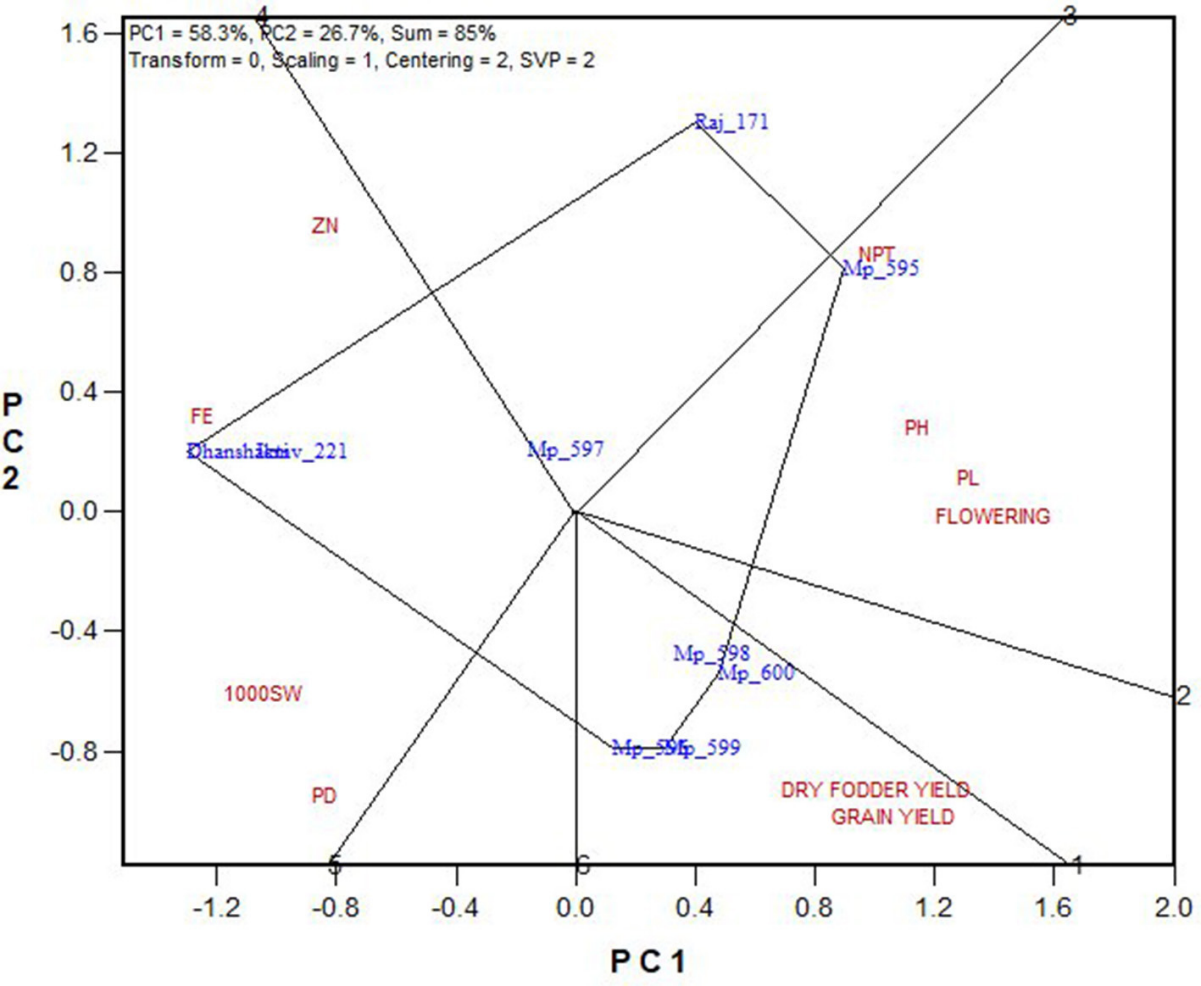
Polygon view of GT biplot indicating which is the best genotype for the target traits.

“Which is best for what” analysis of the genotype × trait biplot helped to compare genotypes on the basis of multiple traits, and to identify genotypes superior for a particular trait (Figure 7). The biplot indicates that MP 595 was the best for PHT, PL, NPT, and late flowering. OPVs MP 596, MP 598, MP 599, and Mp 600 were better for GY and DFY; while Dhanshakti and ICMV 221 performed well for early flowering, grain Fe and Zn contents.

## Discussion

Pearl millet is grown in arid and highly arid tracts of India under minimal or no inputs with hybrids and OPVs as cultivar options. Over a period of time, 61 OPVs are released. This figure is very low compared to the hybrids available for the commercialization of pearl millet. However, few OPVs, such as ICTP 8203 and ICMV 221, are popularly grown, owing to their resilience to marginal conditions and good grain quality apart from reasonable yield levels. Though the OPVs have not received the attention they deserve, few research centers are continuing to develop OPVs with improved yield. Determination of the performance of improved OPVs to compare with that of the popular OPVs for yield and quality traits, and identification of suitable OPVs for target locations are required for focusing research efforts. The 20 multi-location testing sites across India used for the study on pearl millet are handled by AICRP and represent diverse pearl millet production ecosystems. GGE biplot, effectively used in many crops, has been used to analyze MET data and interpret complex GEI (Yan, 2001; Yan and Tinker, 2006), and to obtain high yielding and stable cultivars, derive the relationship among the environments, identify an ideal environment besides “which won-where,” and delineate mega-environments among the testing locations (Yan et al., 2007). We have studied the GEI among nine pearl millet OPVs (six new and three popular varieties) across 20 locations performing GGE biplot analysis. In the combined data from the current study, environment or location contributed 48–91% of the variation in the data, while the contribution of genotype is from 8 to 66% for 10 traits. The interaction of genotype with location is less (1–4%) though significant (Table 3). Gauch and Zobel (1997) reported that normally in MET data, environment accounts for about 80% of the total variation. In barley MET data, environment accounted for as high as 76.7% (Jalata, 2011). Similar trends are observed in proso millet with environment contributing up to 85% (Pan-pan et al., 2016) and up to 82% in sunflower (Santos et al., 2019). However, Tefera (2018) reported a moderate 51.6% of variation being explained by environment in soybean MET data, and Krishnamurthy et al. (2017) reported 40.5% in rice MET data. In this study, GL explained a lesser proportion of the variation than G alone. This explains the lesser fluctuations among the cultivars with change in environment as they are known for high adaptability and stability. Though low, the significant GE indicates that the genotypes showed varied performances across locations. In the GGE biplot analysis, the complex GEI are simplified in different PCs; and if the first two PCs explain more than 60% of the (G and GL) variability in the data and the combined (G and GL) effect accounts for more than 10% of the total variability, then the biplot adequately approximates the variability in G × E data (Rakshit et al., 2012). In this study, the first two PCs explained 67.8% variation for grain yield and 85.5% for fodder yield. In addition, Table 3 indicates that G and GL together accounted for 8 and 14% of total variability for grain and fodder yields, respectively. Thus, the graphical representation of the biplots can be used for deriving stable and ideal genotypes and ideal environments.

### Ideal Genotypes

A genotype is considered ideal if it has a high mean yield and less variable across locations and seasons. The quality of the data could be considered quite reliable because of moderate to high broad-sense heritability (73–98%) over locations (Table 3). It is evident from Figure 3A that the highest grain yielders, MP596 and MP 599, are moderately stable, while MP 600 is comparatively a good yielder with high stability. For fodder yield, MP 599 and MP 600 are good yielders with MP 599 having high stability (Figure 3B). The new OPVs performed exceptionally well for mean performance and stability compared with proven and popularly cultivated OPVs, ICTP 8203, ICMV 221, and Raj171 (Figures 3A,B). It is a known fact that the OPVs in pearl millet would have more stable yields, are more widely adapted than hybrids, and are less vulnerable to pests and diseases (Charyulu et al., 2014). Improving the OPVs for grain yield indirectly influences the income sustainability of farmers. Variety MP 600, though highly stable for GY, showed less stability for DFY. Similarly, MP 599 was highly stable for DFY and comparatively less stable for GY. A genotype showing stability for a trait may not necessarily be stable for other traits. As different traits are governed by a different set of genes and the environment influences the overall cumulative expression of different sets of genes, the genotypes vary with yield and stability. Similar observations have been reported by Rakshit et al. (2012) with sorghum. However, other OPVs have followed similar trends in yield and stability for grain and fodder yields. As grain yield and fodder yields are more preferred traits in OPVs by farmers, OPVs MP 599 and MP 600 can be recommended for cultivation across all regions and are identified as ideal genotypes for recommendation to farmers.

### Ideal Environments

The “ideal” test environment is that which is most discriminating (brings out the differences among the genotypes) and most representative (represents the target region). Discrimination ability and representativeness of a location can be viewed conveniently from the biplot. The environment with a longer vector and the smallest angle with an ideal environment are identified as a perfect test location in terms of being more discriminating and most representative of overall locations (Pan-pan et al., 2016). Locations Ananthapuramu, Gurugram, Durgapura, Mandor, and Coimbatore, with more vector lengths, are more discriminating. The near average locations, namely, Ananthapuramu and New Delhi, are more representative and are suitable for selecting more adapted genotypes. On the other hand, Durgapura and Gurugram, being discriminating and non-representative, are useful for selecting specifically adapted genotypes. With the advantage of such graphical representation, where a generally adapted environment and a specific environment can be identified, cultivar choices and breeding schemes can be made. Similar views are put forth by Jalata (2011) and Rakshit et al. (2012). Closer relationships between the test environments indicated that the same information could be obtained from fewer environments. Thus, for initial testing, similar environments may be removed in future multi-location testing of pearl millet cultivars. This also ensures the optimal allocation of scarce resources while formulating MLTs. The absence of wide obtuse angles between environment vectors (Figure 4) indicates that there were no negative correlations among the test environments, suggesting the absence of a strong crossover GEI across locations for grain and fodder yields, as suggested by Yan and Tinker (2006). This indicated that genotypes performing better in an environment would also be performing in the same direction in another environment, which means that ranking of genotype does not change from location to location. Even though a mixture of crossover and non-crossover types of GEI in MET data is of very common occurrence (Rao et al., 2011), the data from this study did not show a crossover type of interaction, as the genotypes included in this study are OPVs with inherent resilience, and the check varieties are proven for stability and popularly grown by farmers. Stability is also a response to the environment due to the combined properties of their gene combinations. Being more discriminative and representative among all the testing locations, Ananthapuramu is the ideal environment. Also, in this ideal testing environment, OPVs MP 596, MP 599, and MP 600 have high grain yield.

“Which-won-where” is the most attractive feature of the GGE biplot, which graphically addresses crossover GEI, mega-environment differentiation, specific adaptation, etc., and is widely used by several researchers on many crops (Gauch and Zobel, 1997; Yan and Tinker, 2006; Rao et al., 2011, Krishnamurthy et al., 2017, Jadhav et al., 2019, Pan-pan et al., 2016). Based on this graphical representation, for grain yield, the testing locations were partitioned into three mega environments (ME). ME1 was represented by Vijayapura, Ludhiana, Dhule, Aurangabad, Morena, Palem, Jamnagar, Malnoor, Hisar, Jamnagar, New Delhi, Ananthapuramu, Mandor, Durgapura, and Coimbatore, with MP 595, MP 596, and MP 599 as winning genotypes. For ME1, Ananthapuramu can be selected as the most representative environment. ME2 consisted of locations Vijayanagaram, Perumallapalle, and Niphad, with MP 597 as the winning genotype and Perumallapalle as the most representative environment. ME3 consisted of the Bikaner and Gurugram locations, for which no genotype performed better. Gurugram can be selected from ME3, as it is more discriminative than Bikaner (Figure 4), for initial testing of cultivars and planning of breeding activities. However, this mega-environment pattern needs to be verified through multi-year and multi-environment trials (Rakshit et al., 2012), as proposed for wheat (Yan et al., 2000). The advantage for grain and fodder yields in the new OPVs compared to traditionally grown check varieties can be clearly observed from the study. Hence, more efforts can be targeted for new OPV development, as the advantage of OPVs in resource-constrained and environment-challenged areas cannot be ignored.

### Grain Quality

Genetic variance was very high for grain Fe content and moderate for Zn content, indicating less influence of interaction with the environment on the expression of these traits. The checks had higher grain Fe and Zn content than the new OPVs, while the new OPVs had higher grain and fodder yields. This can also be seen from the trait associations where there was a significantly negative correlation between grain yield and quality traits. However, the new OPVs meet the minimal requirement for grain Fe content of 42 ppm and for grain Zn content of 32 ppm, which is fixed by the AICRP on pearl millet. Though the Fe-rich variety, Dhanshakti, has higher levels of Fe and Zn contents, the check variety, ICMV 221, has comparative levels of Fe and Zn and more stable for grain quality. The grains harvested from the A-zone locations (more from New Delhi, Hisar, and Bikaner) have higher Fe and Zn contents compared with those from the B-zone locations (Dhule, Aurangabad, and Malnoor). The grain Fe and Zn contents seem to be under genetic control and show very less interaction with the environment. Similar to other traits, the influence of location is very high, and the northern part of India has higher levels than the southern India locations. Thus, while fixing the minimal levels of Fe and Zn contents in the grain for varietal release, this aspect has to be considered.

### Genotype × Trait Associations

From the trait relationships and GT biplot, it can be observed that the grain quality traits (grain Fe and Zn contents) are highly related and associated with seed size and panicle density along with early flowering and with check varieties ICMV, 221, and Dhanshakti being promising for them. Most of the tested varieties performed best for the economically important traits, GY and DFY, which are highly correlated with each other, poorly correlated with agronomic traits, and negatively correlated with grain quality traits. For other agronomic traits, MP 595 has performed well. Though the grain and dry fodder yields are the most important traits in any crop improvement program, the pearl millet cultivars grown in arid regions should have early flowering and high tillering to sustain harsh climate, apart from yield and quality. The cultivars grown in semi-arid regions require high grain yield contributed through increased panicle length and density in medium to late maturity background. Hence, selection based on correlated response is required to develop a variety that has higher yields in the desired flowering and tillering background along with good grain quality that is not found in a single variety in this study. Thus, this study has identified a wider scope to develop promising OPVs of pearl millet with breeding strategies based on its outputs.

## Conclusion

Analysis of variance showed significant differences among genotypes and locations. The GEI was significant though comparatively less than location and genotype effects as the cultivars used in the study are known for their stability. The study has identified the best varieties suitable for cultivation across 20 locations of pearl millet growing areas in India. OPVs MP 599 and MP 600 are identified as ideal genotypes, because they showed higher grain and fodder yields and stability than other cultivars. The study has also shown that the genotypes with high mean performance and stability for a trait may not show similar performance for another trait. On the other hand, the Ananthapuramu location had the best discrimination and better representativeness than other locations. Therefore, Ananthapuramu is the ideal test site for selecting pearl millet cultivars effectively for adaptation across India while Ananthapuramu, Perumallapalle, and Gurugram can be used as initial testing locations based on this study. G × E interaction had very little influence on grain Fe and Zn contents. Dhanshakti had higher levels of grain Fe and Zn contents, while ICMV 221 was most stable for traits. The check varieties had higher Fe and Zn contents compared with the new OPVs, which is also reflected in the negative correlation of grain yield with grain Fe and Zn contents. Breeding efforts should be directed to break this linkage. However, the new OPVs meet the required levels of 42 ppm Fe and 32 ppm Zn, fixed by AICRP on pearl millet. Breeders can focus on one of these traits as the correlation between grain Fe and Zn content is very high.

## Data Availability Statement

The raw data supporting the conclusions of this article will be made available by the authors, without undue reservation.

## Author Contributions

CS and VK conceived and designed the study. HP, PG, LS, KM, SS, RN, HB, KI, MT, DY, RB, AT, VKT, UK, KS, MS, BA, NA, and MG planned and performed the experiments. PS wrote the manuscript. MG, TN, and VAT revised the manuscript. All the authors have read and approved the final manuscript.

## Conflict of Interest

The authors declare that the research was conducted in the absence of any commercial or financial relationships that could be construed as a potential conflict of interest.
